# Recent Experiences with Tuberculin

**Published:** 1906-01-13

**Authors:** 


### Hospital Clinics.

### RECENT EXPERIENCES WITH TUBERCULIN.

There has for some time past been a recrudescence of interest in the possibilities underlying the tuberculin treatment of tuberculosis, a recrudescence traceable for the most part to the influence of Professor A. E. Wright. It will be remembered that in the early days of its existence tuberculin established a somewhat evil reputation owing to the occasional occurrence of fatalities after its use, the injection of the tuberculin having apparently given a fresh impetus to the virulence of the infection. Professor Wright, after a long period of investigation, has concluded that the evil effects which have on occasion attended the use of tuberculin as a curative agent depend in reality upon indiscretions of dosage. This is easily understood, for until a fairly recent date there existed no standard by which the appropriate dosage at any given moment could be with any certainty forecasted. But by means of the "*opsonic index*," as estimated by Professor Wright's methods it appears to be possible (at the expense of considerable labour and technical skill, it is true) so to apportion the dose as to reduce to a minimum the likelihood of injurious effects, while in competent hands tuberculin-vaccination, under such stringent regulation, has given very gratifying results.

A few weeks ago there were read before the Medical and Chirurgical Society [1] a number of communications dealing with the tuberculin-treatment of tuberculosis under the control of observations upon the opsonic index of the blood. Before proceeding to give an abstract of one of these communications (one which deals with instances of pulmonary disease), it will not be amiss to remind the reader of the broad facts underlying the method of control.

The tuberculin used by most observers in this field of therapeutics is Koch's new tuberculin, the Tuberculin R. This consists of a suspension of dead and triturated tubercle bacilli. It is called T.R. tuberculin (*i.e.* tuberculin ruckstand) in contradistinction from the T.O. tuberculin (*i.e.* oberer tuberculin). The dried bacilli are triturated in distilled water; the whole is then centrifugalised. The supernatant fluid is the T.O., the upper tuberculin. The solid residue is again extracted several times with distilled water; these successive extracts are mixed together, and, holding the ultimate solid residue in suspension, form the T.R. or residual tuberculin. The object of the injections is to create in the infected organism an active and specific immunity against the tubercle bacillus. Variations in the degree of specific immunity are indicated by variations in the opsonic index of the blood, to be presently explained.

Dr. Wright [2] claims to have proved that there exists in normal serum, and more particularly in the sera of persons successfully inoculated against any particular micro-organism, an element which enters into chemical combination with the microbe in question in such a way as to prepare it for phagocytosis. This element he has named an *opsonin*. It is asserted that, apart from the action of the specific opsonin, phagocytosis cannot take place. The extent to which any specific opsonin is present in the blood is susceptible of accurate measurement by observation of the rapidity with which phagocytosis of the corresponding organism takes place. Shortly stated the method is as follows: In a capillary tube are mixed one volume of the patient's serum, one volume of suspended tubercle bacilli, and one volume of corpuscles from normal blood, citrated to prevent clotting. This tube is placed in an incubator at body heat for twenty minutes, at the expiry of which interval film preparations are made. After appropriate staining, the first thirty or forty white corpuscles which come into view are examined and the number of ingested bacilli in each corpuscle duly noted. The "phagocyte count" is arrived at by adding together the total of ingested bacteria and dividing this figure by the number of cells examined. A similar count is then made from the contents of a tube similarly prepared in every particular, except that instead of the patient's serum there is added one volume of serum from a healthy man. The phagocyte count of the normal blood is taken as unity, and the ratio in which the phagocyte count of the patient's blood stands to the former is called the "*opsonic index*." It is necessary to observe that every injection of T.R. is followed very soon by a phase of reduced resistance—what Professor Wright calls the "negative phase." This negative phase, the duration of which is variable, is followed by a positive phase, or phase of gradually increased resistance to the specific organism; the positive phase results in a gradual decline, but the object of the treatment is attained if at the termination of these undulations the index remains at a higher level than that which marked the period previous to the treatment. The evil results of past experiments with tuberculin are probably to be credited to the repetition of injections during negative phases, by which means a cumulative action in the direction of diminished resistance resulted too often in a general dissemination of the infecting agent.

With this somewhat copious but necessary introduction we may reproduce the chief points of the communication of Drs. David Lawson and Ian Stewart upon the administration of T.R. in cases of pulmonary tuberculosis. The patients upon whom the observations recorded in the paper were carried out were for the most part subjects of a sub-acute or chronic form of the disease, and were not febrile at the time. The authors have set themselves four questions, which are considered in order.

Firstly: Does any difference exist in the opsonic indices of the blood in (*a*) healthy persons; (*b*) in persons who have suffered from tuberculosis and are now cured; and (*c*) in persons who are suffering from pulmonary tuberculosis (not acute)?

If so, can the facts be turned to any account in the diagnosis of the disease?

The observations of the authors, backed by the independent observations of Dr. Bulloch, demonstrate that while a marked degree of variation may be observed in the opsonic indices of health, it is a variation within clearly defined limits, namely, between 0.9 and 1.2. They consider it proven that in healthy people the opsonic index in the matter of the tubercle bacillus does not transgress these limits.

The second branch of this question led to the examination of twenty-five patients who had passed through the hands of the writers, and who fulfilled all the known tests of cure. In exceedingly few of these cases did the opsonic index approach the minimum figure of health. Notwithstanding their apparent good health the blood of these subjects is singularly deficient in protective substances in relation to the tubercle bacillus.

For the purposes of the third sub-heading twenty-five patients were examined who at the time of examination were undoubtedly suffering from pulmonary tuberculosis, though not in an acute form. Among these twenty-five there were four only whose opsonic indices were high enough to come within the limits of healthy variation.

The conclusion, therefore, is that those who have, and those who have had, active pulmonary tuberculosis will present an opsonic index almost invariably lower than that of a healthy subject. Professor Wright has suggested that the opsonic index may be used as a means of diagnosis. The authors agree with him that in a dubious case an opsonic index either exceeding or falling below the limit of variation in health is to be regarded as highly suggestive of tuberculous invasion.

Secondly: Assuming that the above classes of person have been inoculated with small doses of tuberculin, is such inoculation followed by a difference in the behaviour of the opsonic index in each class? If so, can this fact be utilised in connection with the diagnosis of the disease?

The answer to this question involves the inoculation of healthy subjects. Consequently the data are few. In the case of four healthy men the opsonic index rose immediately upon the injection—a circumstance remarkably in contrast with the almost constant occurrence of a negative phase in the case of persons previously infected with the tubercle bacillus. The authors consider that if more extended experiments confirm this observation, examination of the effect of a small injection of tuberculin upon the opsonic index of a suspected person will greatly assist the diagnosis of tuberculous disease.

Thirdly: Is there any means by which the presence and duration of the negative phase of the blood can be reliably determined other than the laborious method of making daily observations of the opsonic index? In the attempt to answer this question the authors made many careful examinations of the effect of a negative phase (as estimated by the opsonic index) upon the respiration, pulse, and temperature. Their conclusion is that none of these afford any criterion of the duration of the negative phase.

Fourthly: To what practical use, if any, can the facts so far elicited be put in the treatment of pulmonary tuberculosis?

It is assumed as a correct proposition that the injection of tuberculin during a negative phase will reinforce the negative phase. In a series of 120 patients a negative phase followed inoculation in all but fifteen instances. The negative phase varied in duration, but it is pointed out that a routine inoculation—say twice weekly—would have resulted in reinforcement of the negative phase in 46 instances. Such reinforcement would tend to repeat the catastrophes of the early days of tuberculin; a fact which makes it clear that in daily observation of the opsonic index, and in that alone, have we a guide to prevent us from reinoculating a patient at a dangerous moment. Finally, investigations conducted upon numbers of patients who, after thorough trial of sanatoria in many parts of the world, had arrived at a more or less stationary condition show that by means of carefully conducted inoculations, checked by consideration of the opsonic index, a very definite rise in the specific resistance to the tubercle bacillus can be attained.

[1] Nov. 28th, 1905. [2] Nov. 9th, 1904, Clin. Jour.

